# Occupational diisocyanate asthma of a female professional driver – the importance of allergological diagnostics 

**DOI:** 10.5414/ALX02133E

**Published:** 2020-12-08

**Authors:** Christian Eisenhawer, Monika Raulf, Rolf Merget

**Affiliations:** Institute for Prevention and Occupational Medicine of the German Social Accident Insurance (IPA), Ruhr University Bochum, Bochum, Germany

**Keywords:** diisocyanates, occupational asthma, inhalation test, sensitization, prevention

## Abstract

Diisocyanates continue to be one of the most frequent causes of occupational asthma worldwide. They are still indispensable in industrial use as components of coatings, glues, and polyurethane foams. In Germany, respiratory diseases due to diisocyanates can be compensated by the statutory accident insurance (according to BK-Nr. 1315). The present case report shows a rare case of sensitization against diisocyanates verified by skin prick test and serological testing. Due to these findings, a modified inhalation test with an extremely low initial diisocyanate concentration in the laboratory was performed, and a positive reaction could be detected already after an extremely low diisocyanate concentration. In addition, increases of fractional exhaled nitric oxide (FeNO) and eosinophils in induced sputum after inhalation testing were seen. The present case constellation underlines the particular importance of allergological tests for diagnostic clarification of the diagnosis of diisocyanate asthma.

**German version published in Allergologie, Vol. 43, No. 3/2020, pp. 89-93**

## Introduction 

Exposure to diisocyanate vapors and aerosols can lead to irritations of the skin, mucosa, and airways. “Isocyanate asthma” in a narrower sense is an immunological asthma triggered by diisocyanates. From a chemical point of view, diisocyanates are isocyanic acid esters, highly reactive compounds with one or more immunologically relevant reactive NCO groups. Monoisocyanates have a strong irritative potential and can cause reactive airway dysfunction syndrome (RADS); an immunological effect has not been described for this group of substances. In rare cases, toxic pulmonary edema may develop after extremely high exposure. In Germany, respiratory diseases due to diisocyanates can be compensated by the statutory accident insurance (according to occupational disease no. (BK-Nr.) 1315). The number of confirmed cases has been relatively stable over the past few years (2016 (n = 59), 2017 (n = 44), 2018 (n = 50)). 

The effect mechanism in immunological asthma caused by diisocyanates has not yet been fully clarified, but it is considered a certainty that dose-effect relationships exist, and, as with other forms of asthma, there is an inflammatory reaction of the airways with an increase in Th2/Treg lymphocytes and eosinophilic granulocytes [1, 2, 7]. Specific IgE antibodies are only rarely detected. IgG antibodies can serve as markers of exposure and as a diagnostic criterion for the rare diisocyanate-related hypersensitivity pneumonitis (HP); there have also been reports of combined asthma and HP. It is often difficult to distinguish between irritant and immunologic effects, particularly when inhalation testing is impossible. General symptoms of type-1 sensitization, like rhinitis or conjunctivitis, are relatively rare. In the case of IgE-mediated sensitization, even low concentrations in the range of 1 pbb can trigger asthmatic reactions [4, 8]. 

The diagnosis of diisocyanate asthma, and particularly the confirmation of an exposure-related obstructive airway disease, is still challenging. For diagnostic workup, non-invasive procedures like serial measurements of fractional exhaled nitric oxide (FeNO), bronchial hyperresponsiveness or eosinophils in induced sputum as well as skin prick testing and the detection of specific IgE antibodies in serum are available. In most cases, inhalation tests with diisocyanates are required to detect an occupational disease but should be used with caution, especially if there are signs of diisocyanate sensitization. Diisocyanate-specific IgE and/or IgG antibodies are detectable in serum in only 5 – 20% of exposed persons. In one of our studies, no specific IgE antibodies could be detected in 93 diisocyanate-exposed subjects suspected of having an occupational respiratory disease; however, in 3 cases, marked immediate reactions with a significant FEV_1_ reduction were seen, which appeared to be more severe than the late-type reactions [6]. When antibodies are negative, a specific inhalation test can be useful. However, standardized inhalation tests are complex and have been established in only a few specialized centers. In the presence of work-related symptoms, non-invasive procedures before and after inhalation the test, such as serial FeNO measurements, methacholine (MCH) tests and assessment of eosinophils in induced sputum can increase sensitivity [9]. The following case report aims to underline the importance of allergological diagnostic workup in the case of a pronounced specific immunological reaction to diisocyanates. 

## Case report and results 

A 60-year-old female professional driver first presented in November 2018. She reported repeated exposure to methylene diphenyl diisocyanate (MDI) at the loading point during the transport of chemicals. From May 2017 onwards, she progressively developed cough, extreme shortness of breath, and sweating so that the treating physicians prescribed asthma therapy. A leakage in October 2017 caused high dermal and inhalative exposure, which led to erythema, scaling of the skin, strong cough and dyspnea. Since then, cough occurred after even the slightest MDI exposure. Currently, she only transports toluene-2,4-diisocyanate (TDI) with the use of respiratory protection, and is symptom-free. At the time of examination, when drugs affecting the airways were not used, no respiratory problems, including those related to bronchial hyperresponsiveness, were reported. The patient’s allergy history was unremarkable, without pre-existing airway disease. Treatment with long-acting β-2 agonists was last used 3 weeks before diagnosis. At the time of examination she was free of infections and symptoms. Spirometry and body plethysmography showed mild obstruction, MCH testing demonstrated severe bronchial hyperresponsiveness. Skin prick test using a conjugate of human serum albumin (HSA) and MDI had already detected a sensitization (wheal 6 mm, erythema 20 mm). At a total IgE concentration of 144 kU/L, MDI-specific IgE (CAP class 3) and MDI-specific IgG antibodies (higher concentration than the maximum value in the reference collective of 121 healthy subjects) could be detected (Table 1) [10]. Based on these findings, a modified short-term inhalation test with 3 ppb MDI over 1 minute was carried out in deviation from the standard exposure protocol (Figure 2). Effect parameters were FEV_1_, specific airway resistance, FeNO, and eosinophilic granulocytes in the induced sputum. In the lung function test 20 minutes after exposure, the positivity criteria were met, and at the day after the examination, a FeNO increase from 12 to 26 ppb was seen. Examination of the sputum showed a percentage increase (from 23 to 31%) and an absolute increase (from 3.6 × 10^6^ to 4.1 × 10^6^) of eosinophilic granulocytes after the inhalation test (Figure 3). This constellation of findings indicates a sensitization to MDI and, due to the work-related complaints in the absence of relevant confounders, the presence of occupational diisocyanate asthma. The recognition and compensation as an occupational disease was recommended. No indications for HP were present. 

## Discussion and conclusion 

Diisocyanates continue to be among the most frequent causes of occupational asthma worldwide [3]. The early detection of the first symptoms and an early avoidance of exposure are of great importance for the further course of the disease [5]. Once sensitization has occurred, it is usually not possible to continue employment even at workplaces with low exposure, since an asthmatic reaction can be triggered even at extremely low diisocyanate concentrations [4]. Allergological test procedures and inhalation tests are used to detect immunologically mediated diisocyanate asthma and are applied in expert examinations as part of assessment procedures for occupational diseases. 

**Figure 1.**
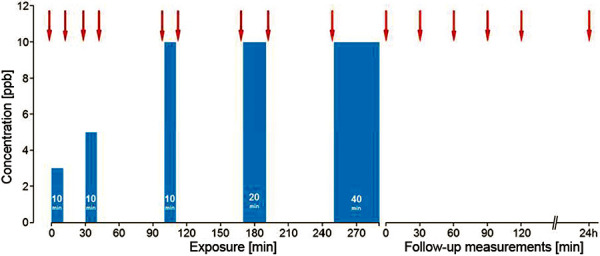
Standard diisocyanate inhalation test protocol. Blue columns represent diisocyanate testing, red arrows mark the time points of lung function measurements. ppb = parts per billion.

**Figure 2.**
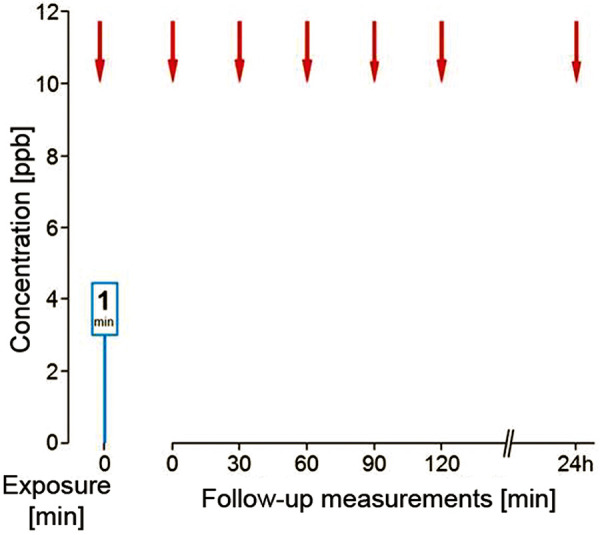
Modified diisocyanate inhalation test protocol. Blue columns represent diisocyanate testing, red arrows mark the time points of lung function measurements. ppb = parts per billion.

**Figure 3.**
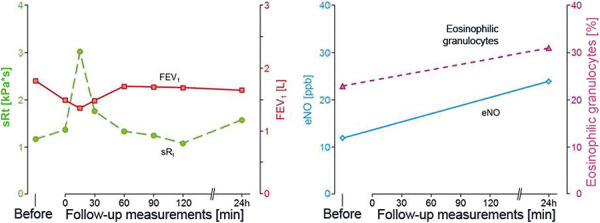
Effect parameters before, 20 minutes, and 24 hours after inhalation testing. sRt = specific airway resistance; FEV_1_ = forced expiratory volume in 1 second; eNO = exhaled nitric oxide; ppb = parts per billion.

The present case report shows a very rare case where sensitization against diisocyanates could be verified. The cause was not chronic exposure to diisocyanate but several accidental high exposures. Both, the skin prick test with HSA-MDI conjugate as well as the serological diagnostic work-up showed signs of an IgE-mediated immunologic reaction to MDI. Due to the relatively low increased total IgE concentration (144 kU/L), a non-specific binding was assumed with regard to the serological findings. This was clearly confirmed by the negative findings for maltose-binding protein (MPB) and HSA. Thus, a high specificity of the specific IgE result can be assumed in the present case. However, since sensitization after high-dose exposure has been rarely described in the literature, we decided to carry out a whole body inhalation test with MDI, especially for preventive reasons. Due to the proven sensitization, the usual inhalation test scheme (Figure 1) [11] was modified, and a positive reaction occurred even after an extremely low diisocyanate concentration (Figure 2). In the follow-up examinations, a positive result could be documented in the serial measurements of FeNO and eosinophilic granulocytes in induced sputum (Figure 3). Positive inhalation tests have been generally described in the literature also without the detection of type I sensitization to diisocyanates [4]. If sensitization is known, as in the present case, the indication for inhalation testing should be carefully checked in order to avoid severe asthmatic reactions. Due to the high predictive value at low total IgE levels and the high specificity of diisocyanate-specific IgE antibodies, an assessment of the causal relationship would also be possible without inhalation challenge test if clear workplace-related symptoms were present. 

From a preventive point of view it has to be discussed, whether this patient with proven sensitization to MDI can continue to work with TDI. On the one hand, the patient no longer reported any work-related symptoms, but on the other hand, sensitization to TDI could also been shown. Cross-reactivities between diisocyanates are known from animal experiments. To our knowledge, there are no data on concomitant allergies to two different diisocyanates in humans, and we have never seen such a case. However, the number of patients in whom we have carried out exposure tests with two different diisocyanates is very small. Regarding this patient, we have informed the accident insurance that serial FeNO and FEV_1_ measurements would be an option to further address this question. 

## Acknowledgment 

We thank Silke Maryska and Ursula Meurer for their technical assistance in IgE and IgG diagnostics. For their support during the examination of the patient we thank: Anja Molkenthin, Jennifer Gili, Melanie Ulbrich, and Susann Widmer. 

## Funding 

The study was financed by the German Social Accident Insurance (projects IPA-004 and IPA-14). 

## Conflict of interest 

The authors have no conflict of interest to report. 

**Table 1.** Antigen-specific antibodies in serum. Specific IgE concentrations ≥ 0.35 kU/L are considered positive.

ImmunoCAP		Concentration
Total IgE		144 kU/L
Specific IgE	Isocyanate TDI	3.38 kU/L
	Isocyanate MDI	11.9 kU/L
	Isocyanate HDI	6.86 kU/L
	Maltose-binding protein (MPB)	0.00 kU/L
	Human serum albumin	0.01 kU/L
Specific IgE	Isocyanate TDI	< 2
	Isocyanate MDI	7.06 mg/L
	Isocyanate HDI	< 2
